# Development of a nomogram to predict surgical site infection after open reduction and internal fixation for closed pilon fracture: a prospective single-center study

**DOI:** 10.1186/s13018-023-03598-8

**Published:** 2023-02-15

**Authors:** Lei Xie, Guofeng Liu, Xin Wang, Zixuan Luo, Yansen Li, Xiaomeng Wang, Fengqi Zhang

**Affiliations:** 1 Department of Orthopaedic Surgery, The Third Hospital of Hebei Medical University, No. 139 Ziqiang Road, Shijiazhuang, 050051 Hebei Province People’s Republic of China; 2 The Sixth Department of Orthopaedic Surgery, The HanDan Central Hospital, HanDan, Hebei Province People’s Republic of China

**Keywords:** Predictor, Nomogram, Surgical site infection, Closed pilon fractures, Open reduction, Internal fixation

## Abstract

**Background:**

To explore the risk factors and develop a nomogram in order to predict surgical site infection (SSI) after open reduction and internal fixation (ORIF) for closed pilon fractures (CPF).

**Methods:**

A prospective cohort study with one-year follow-up was carried out in a provincial trauma center. From January 2019 to January 2021, 417 adult patients with CPFs receiving ORIF were enrolled. A Whitney U test or *t* test, Pearson chi-square test, and multiple logistic regression analyses were gradually used for screening the adjusted factors of SSI. A nomogram model was built to predict the risk of SSI, and the concordance index (C-index), the receiver operating characteristic (ROC) curve, calibration curve and decision curve analysis (DCA) were used for evaluating the prediction performance and consistency of the nomogram model. The bootstrap method was employed to test the validity of the nomogram.

**Results:**

The incidence of SSI after ORIF for CPFs was 7.2% (30/417): 4.1% (17/417) of superficial SSIs and 3.1% (13/417) of deep SSIs. The most common pathogenic bacteria were Staphylococcus aureus (36.6%, 11/30). The multivariate analysis showed tourniquet use, longer preoperative stay, lower preoperative albumin (ALB), higher preoperative body mass index (BMI) and hypersensitive C-reactive protein (Hs-CRP) were independent risk factors of SSI. Additionally, the C-index and bootstrap value of the nomogram model were 0.838 and 0.820, respectively. Finally, the calibration curve indicated that the actual diagnosed SSI had good consistency with the predicted probability, and the DCA showed that the nomogram had clinical value.

**Conclusions:**

Tourniquet use, longer preoperative stay, lower preoperative ALB, higher preoperative BMI and Hs-CRP were five independent risk factors of SSI after closed pilon fractures treated by ORIF. These five predictors are shown on the nomogram, with which we may be able to further prevent the CPS patients from SSI.

*Trial registration* NO 2018-026-1, October /24/2018, prospectively registered. The study was registered in October 24, 2018. The study protocol was designed based on the Declaration of Helsinki and admitted by the Institutional Review Board. The ethics committee approved the study on factors related to fracture healing in orthopedic surgery. Data analyzed in the present study were acquired from the patients who underwent open reduction and internal fixation from January 2019 to January 2021.

## Background

The pilon fracture is an intra-articular fracture of the distal tibia, which accounted for approximately 6.37% of total tibial-fibular fractures and 1.03% of the whole-body fractures in Chinese adults [1]. More males than females had the pilon fracture and the most vulnerable age group was 41–45 years. The mechanism of injury is a comminuted fracture of the ankle joint accompanying various degrees of distal extension into the tibial-fibular metaphysis. The pilon fractures are relatively uncommon, but the risk of severe complications and long-term disability appears from time to time [2]. Stable pilon fractures with minimal displacement are usually managed by conservative treatments, while unstable pilon fractures by operative treatments. These operative approaches include: screw fixation, use of a locking compression plate, circular ring external fixation, etc. As for the closed pilon fractures (CPFs), open reduction and internal fixation (ORIF) is the most commonly used surgical procedure. However, overexposure of soft-tissue during the ORIF may secondarily injure the fracture site, which is highly prone to leading to postoperative complications such as infection, ankle traumatic arthritis or fracture malunion [3–5].

In all of the above complications, surgical site infection (SSI) remains an important complication of pilon trauma. The traditional ORIF was first developed by Rüedi and Allgöwer [6], which has been related to significant rates of SSIs and wound dehiscence, ranging from 0–55% [7, 8]. According to the reported literature [9], SSI had increased an average of 10 days of hospital stays, which costed an estimated more than £700 m a year in the UK. Regarding the significant economic burden on health system, approximately half the infection events could be early intervened with the evidence-based prevention models implemented [10]. Thus, the quantification of SSI-related risk factors for patients with CPFs could come up with a cost-effective approach. A few studies have explored the predictions of SSI after closed pilon fracture (CPF) treated by ORIF, but these prediction models are not intuitive and easy to use [11–13]. The primary purpose of the related study was to develop a risk assessment model using multivariate logistic regression based on a combination of routine laboratory blood indicators and clinical symptoms. In the current study, risk factors for postoperative surgical site infections such as body mass index (BMI), operative time, preoperative stay, preoperative albumin (ALB), hypersensitive C-reactive protein (Hs-CRP), red blood cell count, and Glycemic index were identified through regression analysis. We were not able to calculate composite risk of SSI after CPF treated by ORIF, even though individual risk factors were reported.

A nomogram is a kind of calculation chart, which can simplify complex mathematical equation and visualize meaningful variables to achieve comprehensive prediction. Hence, we improved the evaluative performance and achieved higher accuracy by comparing multiple models with nomogram, which was performed for the first time to predict the risk of surgical site infection after open reduction and internal fixation for closed pilon fracture. Thus, we designed a prospective protocol to explore the risk factors related to SSI after CPF treated by ORIF and established a nomogram prediction model. The prospective design was carried out for two purposes: on the one hand, to update the epidemiological characters of SSI after ORIF for CPFs; on the other hand, to re-screen the SSI-related factors and develop a nomogram prediction model. Also, it is clinically significant for surgeons in fully understanding the patient's parameters and assessing the risk of postoperative surgical site infections.

## Materials and methods

### Study design

**Fig. 1:**
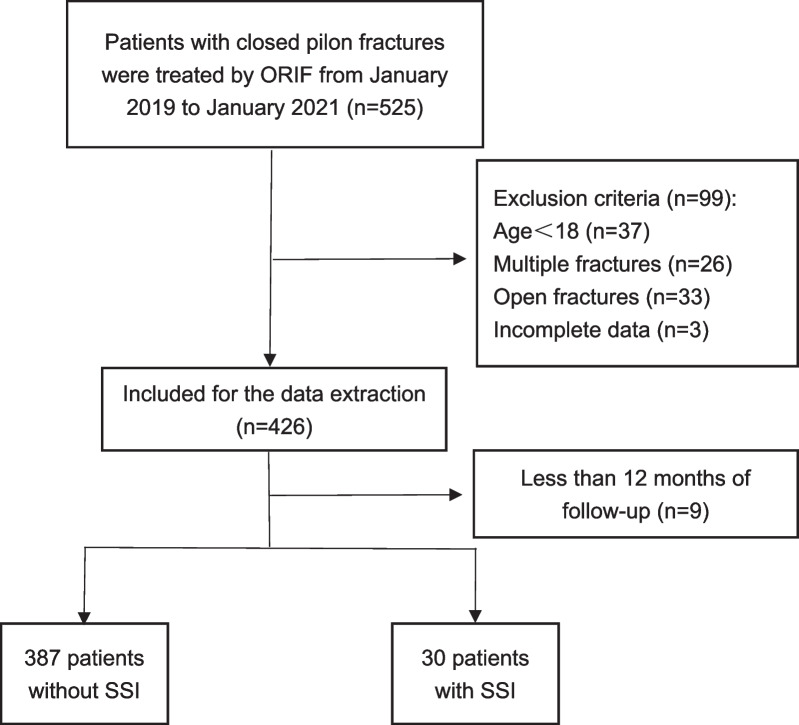
The flow chart for the selection of study participants

The present study was an in-patient’s records-based, prospective cohort trial, and ethical clearance (NO 2018-026-1) was obtained to follow the fracture patient’s data collected in the present study. The protocol was admitted by the Committee on Ethics and the Institutional Review Board from our trauma center. From January 2019 to January 2021, a series of adult patients with CPFs underwent the ORIF were involved. The study was undertaken according to the Declaration of Helsinki and all patients gave written informed consent for the use personal data for research aims. Written informed consent was obtained from each patient to authorize the publication of their data. Exclusion criteria were prescribed as follows: age younger than 18 years (*n* = 37), multiple fracture (*n* = 26), open fracture (*n* = 33), and without complete data (*n* = 3). All enrolled patients were divided into the infection group and the non-infection group according to whether or not infection occurred after operation. The end time point of the following was any evidence of SSI found by the reexaminations during the one-year period post-ORIF. Finally, 417 patients with CPFs performed by ORIF were followed up for a period of one year (Fig. 1).

### SSI diagnosis

According to the definition of the CDC [10], a superficial SSI only involves the skin or subcutaneous tissue of the operation site. A deep SSI was identified if it satisfied one of the following conditions: Infection involves the deep fascia requiring abscess debridement or internal fixation removal. The secretors from the surgical sites were collected for culture and susceptibility tests.

### Data curation and definition of variables

All the participants were followed by three orthopedic surgeons starting from the admission to the hospital. Patients without SSI were followed up by phone calls at 1, 3, 5 and 12 months after discharge. Patients with suspected infection need to receive reexamination and etiological diagnosis. The patients usually have a regular radiological and clinical evaluation every six months after discharge. The specific variables were recorded and classified as the following four groups. First, the demographic variables involved gender, age, body mass index (BMI, kg/m^2^), location(rural or urban), cigarette consumption, alcohol consumption, diabetes mellitus, hypertension and cardiovascular disease. Second, the fracture-related variables involved fracture side(left, right), Rüedi and Allgöwer classification system [6], and injury mechanism. Every patient underwent preoperative assessment with CT scanning. The injury mechanisms were classified as low-energy (slips on the floor), and high-energy (falls from height, traffic crashes and others). Third, the operative variables involved preoperative stay, intraoperative blood loss (mL), operation duration (minutes), anesthetic type (regional or general), tourniquet use, bone grafting use, and American Society of Anesthesiologists (ASA, I-II or III-V) score [14]. Preoperative stay was counted from the first trauma to the ORIF. Four, the laboratory-related variables were collected during one day preoperatively. These biochemistry indices involved white blood cell (WBC), red blood cell (RBC), blood platelet (PLT), albumin (ALB), globulin (GLOB), hemoglobin (HB), hematocrit (HCT), hypersensitive C-reactive protein (Hs-CRP), and blood glucose (GLU).

### Statistical analyses

Statistical analyses were completed using SPSS 24.0. The continuous variables were shown as the mean ± standard deviation (SD). The Shapiro–Wilk test was used to examine distribution normality. Continuous variables between the two groups were analyzed by a Whitney U test or t test based on distribution normality or not. The categorical variables were analyzed with the chi-square test. The variables with significance from the univariate analysis were entered into multiple logistic regression analysis. Finally, a nomogram model was made, and the concordance index (C-index), receiver operating characteristic (ROC) curve, calibration curve and decision curve analysis (DCA) were used to evaluate the prediction performance and consistency of the model. We employed bootstrap method to confirm the performance of the nomogram. Finally, a *p* value < 0.05 was considered as statistically significant.

## Results

### Baseline characteristics of non-SSI and SSI patients

426 patients were reexamined for SSI during the one-year period post-ORIF, in which 9 patients lost to follow-up less than 12 months. Finally, 417 patients with CPFs performed by ORIF were followed up for a period of one year (Fig. 1). The incidence of SSI was 7.2% (30/417): 4.1% (17/417) for superficial SSIs and 3.1% (13/417) for deep SSIs. Staphylococcus aureus was the most common pathogenic bacteria (36.6%, 11/30). As shown in Table 1, for the 417 patients, the average age was 45.6 ± 15.2 years, and BMI was 5.6 ± 4.0 kg/m^2^. Furthermore, BMI (*p* = 0.005) was demonstrated to correlate with SSI.

**Table 1:** Baseline characteristics of non-SSI and SSI patients

Variables	All patients (*n* = 417)	Patients without SSI (*n* = 387)	Patients with SSI (*n* = 30)	*p* value
Gender (males), *n*(%)	267(64.0)	248(64.1)	19(63.3)	0.934^c^
Age (years), mean ± SD	45.6 ± 15.2	45.4 ± 15.2	46.8 ± 14.5	0.586^a^
BMI (kg/m^2^), mean ± SD	25.6 ± 4.0	25.4 ± 3.9	27.7 ± 3.9	0.005^b^*
Location (rural), *n*(%)	218(52.3)	206(53.2)	12(40.0)	0.162^c^
Diabetes mellitus, *n*(%)	17(4.1)	17(4.4)	0(0.0)	0.241^c^
Hypertension, *n*(%)	29(7.0)	27(7.0)	2(6.7)	0.949^c^
Cardiovascular diseases, *n*(%)	32(7.7)	31(8.0)	1(3.3)	0.354^c^
Smoking, *n*(%)	111(26.6)	101(26.1)	10(33.3)	0.388^c^
Alcohol consumption, *n*(%)	112(26.9)	102(26.4)	10(33.3)	0.406^c^
Rüedi and Allgöwer classification				0.786^c^
I, *n*(%)	158(37.9)	148(38.2)	10(33.3)	
II, *n*(%)	197(47.2)	181(46.8)	16(53.3)	
III, *n*(%)	62(14.9)	58(15.0)	4(13.4)	
Mechanism (low energy), *n*(%)	190(45.6)	179(46.3)	11(36.7)	0.310^c^
Side (left), *n*(%)	203(48.7)	188(48.6)	15(50.0)	0.881^c^

*SSI* surgical site infection, *BMI* body mass index

^a^Student t test

^b^Mann–Whitney U test

^c^Pearson Chi-Square test

^*^Indicates significant variable at *p* < 0.05

### Univariate and multivariate analysis

Of the 30 predictive variables listed above, 6 factors were correlated with SSI, which were BMI (*p* = 0.005), preoperative stay (*p* = 0.026), surgical duration (*p* = 0.049), tourniquet use (*p* = 0.003), ALB level (*p* = 0.001) and HCRP level (*p* = 0.001) (Tables 1 and 2). Hence, these 6 factors were included in the multiple logistic regression model. Finally, the multivariate logistic regression analysis demonstrated that tourniquet use (OR = 5.321, *p* = 0.028), longer preoperative stay (OR = 1.112, *p* = 0.022), lower preoperative albumin (ALB) (OR = 0.873, *p* = 0.002), higher body mass index (BMI) (OR = 1.138, *p* = 0.023) and human CRP(hCRP) (OR = 1.012, *p* = 0.000) were independent risk factors for SSI (Table 3). The Hosmer–Lemeshow test showed good fitness (*X*^2^ = 8.086; *p* = 0.425). No collinearity violations occurred for the analysis for the interaction between independent predictors with each other (Tolerance coefficients were all > 0.91, Variance Inflation Factors were all < 1.10).

**Table 2:** Univariate analysis results related to SSI

Variables	All patients (*n* = 417)	Patients without SSI (*n* = 387)	Patients with SSI (*n* = 30)	*p* value
Preoperative stay (days), mean ± SD	5.0 ± 4.0	5.0 ± 4.0	7.0 ± 5.0	0.026^b^
ASA				0.889^c^
I–II, *n*(%)	269(64.5)	250(64.6)	19(63.3)	
III–V, *n*(%)	148(35.5)	137(35.4)	11(36.7)	
Anesthesia type				0.838^c^
Regional, *n*(%)	225(51.8)	210(51.9)	15(50.0)	
General, *n*(%)	201(48.2)	186(48.1)	15(50.0)	
Tourniquet use, *n*(%)	289(69.3)	261(67.4)	28(93.3)	0.003^c^*
Bone grafting use, *n*(%)	24(5.8)	22(5.7)	2(6.7)	0.824^c^
Intraoperative blood loss (ml), mean ± SD	140.0 ± 98.1	139.0 ± 98.0	162.0 ± 96.0	0.076^b^
Surgical duration (minutes), mean ± SD	120.0 ± 38.7	119.0 ± 38.0	135.0 ± 44.0	0.049^b^
RBC (10^12^/L), mean ± SD	3.5 ± 0.6	3.5 ± 0.6	3.3 ± 0.6	0.174^b^
WBC (10^9^/L), mean ± SD	9.8 ± 3.8	9.7 ± 3.7	11.2 ± 5.3	0.247^b^
PLT (10^9^/L), mean ± SD	278.0 ± 114.2	276.0 ± 112.9	300.4 ± 127.9	0.335^b^
HB(g/L), mean ± SD	116.8 ± 13.1	116.6 ± 13.1	118.8 ± 12.8	0.316^b^
HCT(%), mean ± SD	34.6 ± 3.7	34.6 ± 3.7	35.1 ± 3.7	0.393^b^
ALB(g/L), mean ± SD	34.4 ± 5.4	34.7 ± 5.3	30.6 ± 5.2	0.001^a^*
GLOB (g/L), mean ± SD	24.4 ± 4.9	24.4 ± 4.9	25.5 ± 4.7	0.178^a^
HCRP(mg/L), mean ± SD	58.8 ± 56.5	55.2 ± 53.9	102.6 ± 69.6	0.001^b^*
GLU (mmol/L), mean ± SD	6.8 ± 1.8	6.7 ± 1.8	7.0 ± 2.1	0.655^b^

*SSI* surgical site infection, *ASA* American Society of Anesthesiologists, *RBC* red blood cell, *WBC* white blood cell, *PLT* platelet, *ALB* albumin, *GLOB* globulin, *HB* hemoglobin, *Hct* hematocrit, *HCRP* hypersensitive C-reactive protein*, GLU* glucose

^a^Student t test

^b^Mann–Whitney U test

^c^Pearson Chi-Square test

^*^Indicates significant variable at *p* < 0.05

**Table 3:** Multivariate analysis of factors associated with SSI after ORIF

Variables	Odds ratio	95% CI	*p* value
Preoperative stay (days)	1.112	1.015–1.217	0.022
BMI (kg/m^2^)	1.138	1.018–1.272	0.023
Tourniquet use	5.321	1.193–23.727	0.028
ALB(g/L)	0.873	0.803–0.949	0.002
HCRP(mg/L)	1.012	1.005–1.018	0.000

*SSI* surgical site infection, *ORIF* open reduction and internal fixation, *CI* confidence interval, *BMI* body mass index, *ALB* albumin, *HCRP* hypersensitive C-reactive protein

### Development and validation of a SSI nomogram

**Fig. 2:**
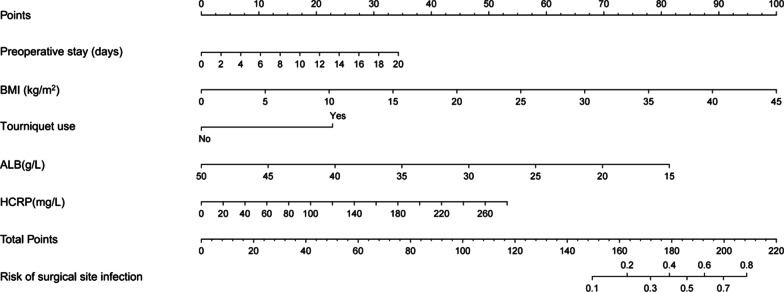
Nomogram for predicting SSI after closed pilon fractures treated by open reduction and internal fixation. BMI body mass index, ALB albumin, HCRP hypersensitive C-reactive protein

**Fig. 3:**
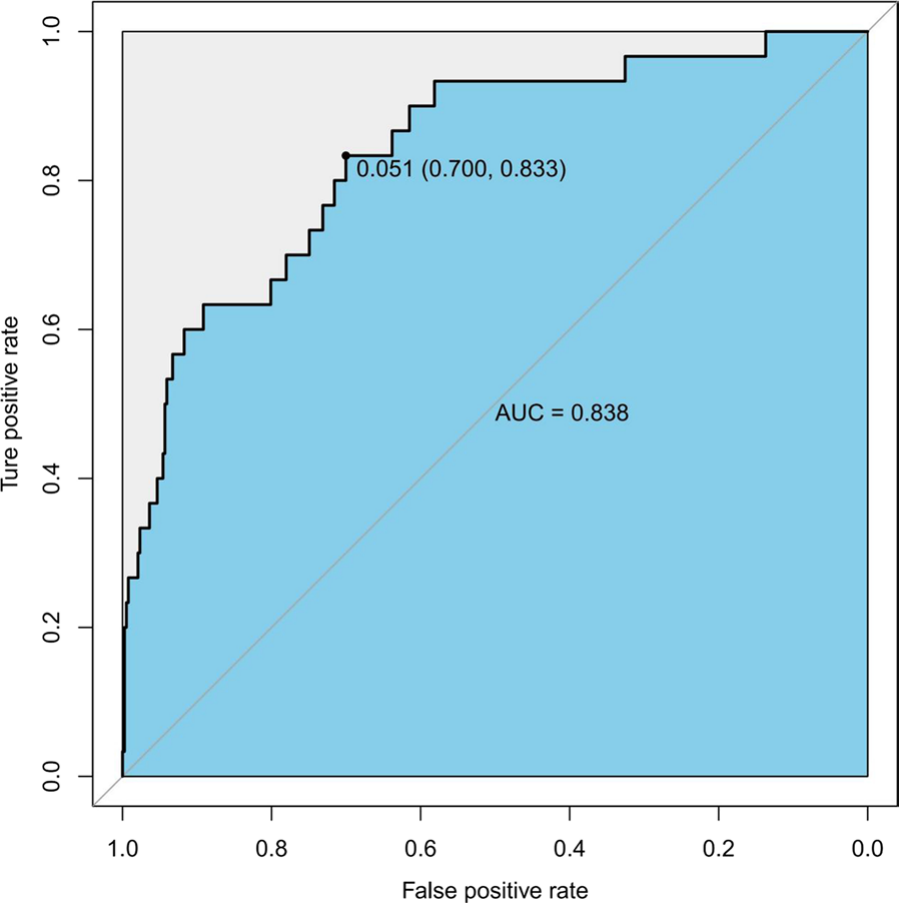
The ROC analysis for the predictive model. *AUC* area under the curve

**Fig. 4:**
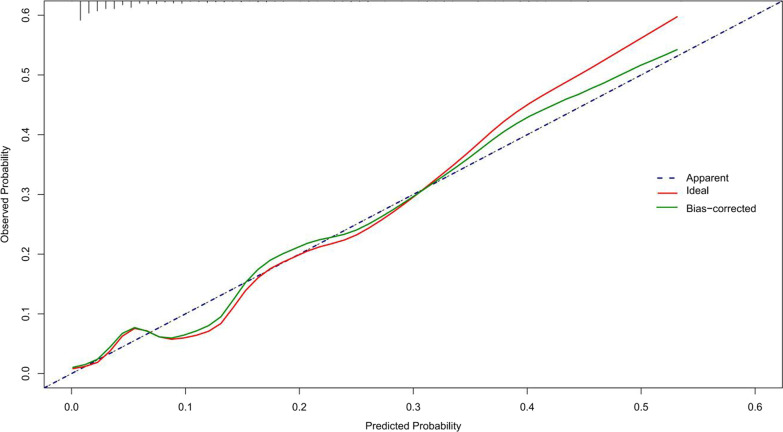
The calibration curve indicated good consistency between the actual diagnosed surgical site infection and the predicted probability

**Fig. 5:**
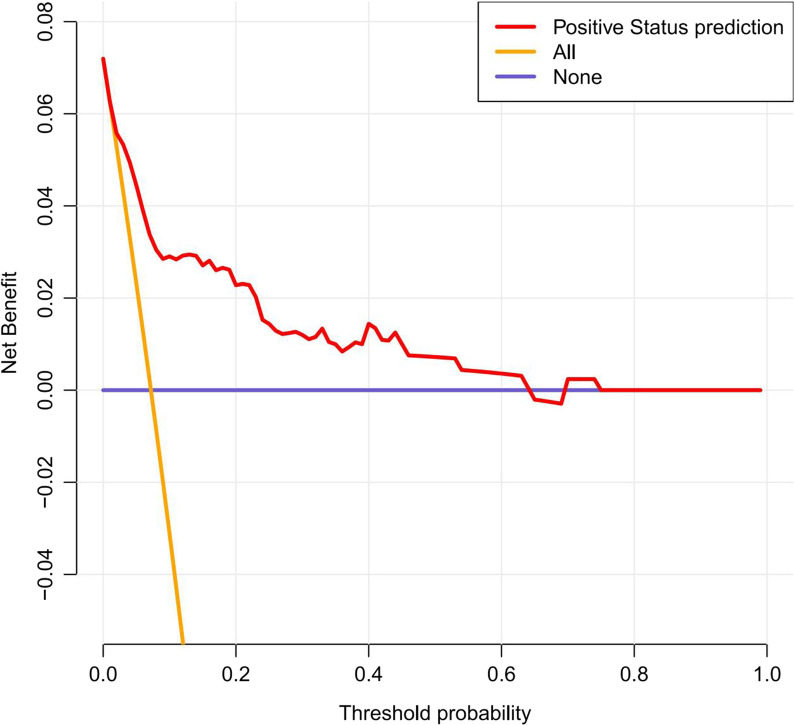
Decision curve analysis for the surgical site infection nomogram

Using the independent risk factors obtained from the multivariable logistic regression analyses, we constructed a nomogram to predict SSI (Fig. 2). By adding individual scores of each predictor in the nomogram, the total score was obtained and used to calculate the corresponding SSI probability. In addition, our analyses showed high predictive accuracy and discrimination of the model,

with a C-index of 0.838 (95% CI 0.760–0.916) and an AUC of 0.820 (Fig. 3). Besides, the calibration curve of the nomogram demonstrated good consistency between the actual diagnosed SSI and the predicted probability (Fig. 4). Similarly, the nomogram DCA indicated that the model could be an excellent prediction tool for SSI after internal fixation of patients with CPFs (Fig. 5).

## Discussion

In the present study of 417 patients, the overall infection rate after ORIF for CPFs was 7.2% with one-year follow-up. It was similar to the result from a meta-analysis involving 10 studies of 8103 cases, in which the incidence of SSI was 7.19% [15]. McCann et al. [16] reported that the most frequent postoperative complication in their study was superficial infection, affecting 7 of 49 pilon fracture patients (14%). Bourne et al. [17] indicated an infection rate of 13% in their high-energy group of 42 fracture series. However, Dillin and Slabaugh [18] showed a higher infection rate up to 55% of 11 high-energy compression pilon fractures, so they recommend ORIF only for low-energy rotational injuries. After adjusting for confounding variables, longer preoperative hospital stay, higher body mass index (BMI), tourniquet use, lower preoperative albumin (ALB), and lower hypersensitive C-reactive protein(Hs-CRP) were associated with post-ORIF SSI in CPFs independent risk factors.

Longer preoperative hospital stay is a well-established risk factor of SSIs following orthopedic surgery [19]. Recent studies have shown that operative time was not associated with wound complications, and that delaying surgery may increase the risk of infection [20, 21]. Two-stage open reduction and internal fixation were considered as a method to protect soft tissue and reduce the infection rate. However, this approach resulted in prolonged hospital stays and lack of anatomical reduction due to surgical delay [22]. Our data suggested that delayed ORIF for closed pilon fracture was associated with an increased risk of SSI, which was consistent with previous reports [19]. A better understanding of psychosocial and socioeconomic factors was critical to identifying opportunities for further intervention to reduce patient morbidity and healthcare costs [12]. In addition to increasing the financial burden by prolonging hospital stays or treatment periods, there was also the concern that delaying surgery may increase the risk of complications such as infection or ankle stiffness. These issues have led some surgeons to consider primary ORIF for pilon fractures. White treated 95 patients with pilon fractures using primary ORIF, and 88% of patients underwent surgery within 48 h of injury. Anatomical reduction was performed in 90% of patients. Finally, six patients developed deep infection and dehiscence requiring debridement [23].

Several studies have proved an association between higher BMI and postoperative infection [24, 25]. Xiaochu Yu et al. [24] also found that obese patients undergoing acetabular fracture surgery had an increased rate of wound infection compared with non-obese patients. Patients with BMI over 28 kg/m^2^ were five times more likely to have a wound infection. Wilson's study showed that an obese class III cohort (BMI ≥ 40 kg/m^2^) was related to a high incidence of SSI after total knee arthroplasty. (The odds ratio for superficial SSI was 4.20 and deep SSI was 6.97.) [25] Metabolic syndrome is an accumulated clinical syndrome in patients with obesity, diabetes, hypertension and dyslipidemia. It may lead to a systemic inflammatory state that increases the risk of postoperative complications including SSI. Pravinkumar et al. [26] reported an increased incidence of superficial wound infection in obese patients who opted for general surgery. In addition, Spaine et al. [27] reported that higher BMI was also associated with increased injury severity, which may also lead to an increased incidence of SSI.

Pneumatic tourniquets are frequently used in various surgeries of upper and lower distal limb to improve the surgical field and reduce intraoperative blood loss [28]. However, tourniquet use in total knee arthroplasty had been shown to increase postoperative wound hypoxia, potentially triggering site infection or delaying wound healing [29]. Due to contact with human skin and proximity to the puncture site, blood residues and bacterial contaminations were regularly found on tourniquets. Hence, tourniquets may be a potential source for cross-contamination with potentially harmful pathogens [30]. As hospital-acquired infections (HAIs) cause substantial costs and increase length of hospital stay and mortality. Another study evaluating patients with elective foot and ankle surgery found that with tourniquets longer than 90 min, there was a sevenfold higher chance of developing postoperative infection or wound-healing complications; but tourniquet use itself was not associated with an increased risk [31, 32]. Non-sterile tourniquets are routinely used in orthopedic surgery in our hospital to obtain a bloodless field. Sahu et al. recommend routine treatment of orthopedic tourniquets with a disinfectant, preferably an alcohol-based solution, to reduce the risk of contamination of the surgical field [33].

The lower albumin (ALB) and elevated hypersensitive C-reactive protein(Hs-CRP) level were identified to be associated with increased risk of SSI in our study. It was similar to the SSI-related studies of other fractures, such as ankle fractures and femur fractures [34, 35]. Abnormalities of these two biomarkers were more likely to indicate poor nutritional and systemic inflammatory status, but a stress effect of trauma cannot be ruled out. In contrast, aggressive preoperative nutritional supplementation to achieve normal serum ALB levels had a significant beneficial effect on reducing SSI and other adverse events [36]. Hs-CRP can detect low concentrations of CRP by hypersensitive detection technology and reflect inflammatory state and tissue injury degree. Hs-CRP has been repeatedly discussed as an inexpensive and readily available immune/inflammatory biomarker-driven index and has been shown to predict adverse events in pneumonia [37], trauma [38] or cardiovascular disease [39] even mortality. Hs-CRP is present in the serum of patients with acute inflammation and is secreted by the liver. It is not affected by factors such as age, gender, body temperature, and anemia and is therefore considered to be the preferred indicator to differentiate between bacterial and viral infections [40]. In our study, if a patient had multiple laboratory tests before surgery, the laboratory test closest to the surgery was selected for data analysis. Therefore, our results may be more valuable in predicting the occurrence of SSI compared with the commonly used biomarker assays at admission, taking into account the changes in biomarkers after trauma.

The present study had three significant strengths: Firstly, it was a prospective, longitudinal one-year follow-up study; second, compared with the traditional multiple regression model, our nomogram enabled healthcare workers to conveniently assess the high-risk groups of SSIs postoperatively, so as to make necessary interventions (such as improving the ALB and Hs-CRP of patients before operation, giving no tourniquet as often as possible, and minimizing preoperative stay, etc.); third, it indicated that tourniquet use and Hs-CRP were an independent indicator of SSI after adult CPFs treated by ORIF. But there are limitations that are worth mentioning. Some variables that potentially influence the development of SSI were not included, such as the internal fixation material (titanium or stainless), surgical incision length, comorbidity disorder, bone mineral density(BMD), hemoglobin and lactate dehydrogenase(LDH).

## Conclusion

Tourniquet use, longer preoperative stay, lower preoperative ALB, higher preoperative BMI and Hs-CRP were independent risk factors for SSI after closed pilon fractures treated by ORIF. These five predictors are shown on the nomogram, with which we may be able to further prevent the CPS patients from SSI.

## Data Availability

All the data will be available upon motivated request to the corresponding author of the present paper.

## Abbreviations

BMI: Body mass index
HGB: Hemoglobin
RBC: Red blood cell
WBC: White blood cell
PLT: Platelet
TP: Total protein
ALB: Albumin
ALT: Alanine transaminase
AST: Aspartate transaminase
CRP: C-reactive protein
PT: Prothrombin time
APTT: Activated partial thromboplastin time
FIB: Fibrinogen
ROC: Receiver operating characteristic
CI: Confidence interval
ASA: The American Society of Anesthesiologists

## References

[1] Zhang Y (2016). Clinical epidemiology of orthopedic trauma.

[2] Flett L (2021). A multicentre, randomized, parallel group, superiority study to compare the clinical effectiveness and cost-effectiveness of external frame versus internal locking plate for complete articular pilon fracture fixation in adults. Bone Jt Open.

[3] Spitler CA (2020). What are the risk factors for deep infection in OTA/AO 43C pilon fractures?. J Orthop Trauma.

[4] Yaradilmis YU (2020). The mid-term effects on quality of life and foot functions following pilon fracture. Ulus Travma Acil Cerrahi Derg.

[5] Van der Vliet QMJ (2019). Long-term outcomes after operative treatment for tibial pilon fractures. OTA Int.

[6] Rüedi TP, Allgöwer M (1979). The operative treatment of intra-articular fractures of the lower end of the tibia. Clin Orthop Relat Res.

[7] Ren T (2015). Risk factors for surgical site infection of pilon fractures. Clinics (Sao Paulo).

[8] Rascoe AS (2020). Factors associating with surgical site infection following operative management of malleolar fractures at an urban level 1 trauma center. OTA Int.

[9] Leaper DJ (2004). Surgical site infection—a European perspective of incidence and economic burden. Int Wound J.

[10] Horan TC (1992). CDC definitions of nosocomial surgical site infections, 1992: a modification of CDC definitions of surgical wound infections. Infect Control Hosp Epidemiol.

[11] Daniels NF (2021). Open pilon fracture postoperative outcomes with definitive surgical management options: a systematic review and meta-analysis. Arch Bone Jt Surg.

[12] Zelle BA (2021). Fate of the uninsured ankle fracture: significant delays in treatment result in an increased risk of surgical site infection. J Orthop Trauma.

[13] Benedick A, Audet MA, Vallier HA (2020). The effect of obesity on post-operative complications and functional outcomes after surgical treatment of torsional ankle fracture: a matched cohort study. Injury.

[14] Zumsteg JW (2014). Factors influencing infection rates after open fractures of the radius and/or ulna. J Hand Surg Am.

[15] Shao J (2018). Risk factors for surgical site infection following operative treatment of ankle fractures: a systematic review and meta-analysis. Int J Surg.

[16] McCann PA (2011). Complications of definitive open reduction and internal fixation of pilon fractures of the distal tibia. Int Orthop.

[17] Bourne RB, Rorabeck CH, Macnab J (1983). Intra-articular fractures of the distal tibia: the pilon fracture. J Trauma.

[18] Dillin L, Slabaugh P (1986). Delayed wound healing, infection, and nonunion following open reduction and internal fixation of tibial plafond fractures. J Trauma.

[19] Chen Y (2021). Comparison of complications of early and delayed open reduction and internal fixation for treating pilon fracture: a protocol of systematic review and meta-analysis. PLoS ONE.

[20] Rüedi TP, Allgöwer M (1969). Fractures of the lower end of the tibia into the ankle-joint. Injury.

[21] Riedel MD (2019). Correlation of soft tissue swelling and timing to surgery with acute wound complications for operatively treated ankle and other lower extremity fractures. Foot Ankle Int.

[22] Konrath G (1995). Early versus delayed treatment of severe ankle fractures: a comparison of results. J Orthop Trauma.

[23] Minator Sajjadi M (2018). The outcomes of pilon fracture treatment: primary open reduction and internal fixation versus two-stage approach. Arch Bone Jt Surg.

[24] Yu X (2019). Effect of a risk-stratified intervention strategy on surgical complications: experience from a multicentre prospective study in China. BMJ Open.

[25] Wilson CJ (2018). Surgical site infection in overweight and obese Total Knee Arthroplasty patients. J Orthop.

[26] Pravinkumar E (2003). Obesity in general elective surgery. Lancet.

[27] Spaine LA, Bollen SR (1996). 'The bigger they come …': the relationship between body mass index and severity of ankle fractures. Injury.

[28] Benedick A, Rivera T, Vallier HA (2020). Effect of tourniquet use during ankle fracture fixation on wound healing and infectious complications. Foot Ankle Int.

[29] Clarke MT (2001). Tourniquetinduced wound hypoxia after total knee replacement. J Bone Joint Surg Br.

[30] Grohmann M (2020). Reduced bacterial contamination rates detected on silicone tourniquets compared to conventional tourniquets in clinical routine. BMC Infect Dis.

[31] Wiewiorski M (2015). Risk factors for wound complications in patients after elective orthopedic foot and ankle surgery. Foot Ankle Int.

[32] Blanco JF (2020). Risk factors for periprosthetic joint infection after total knee arthroplasty. Arch Orthop Trauma Surg.

[33] Sahu SK, Tudu B, Mall PK (2015). Microbial colonisation of orthopaedic tourniquets: a potential risk for surgical site infection. Indian J Med Microbiol.

[34] Meng J (2018). Deep surgical site infection after ankle fractures treated by open reduction and internal fixation in adults: A retrospective case-control study. Int Wound J.

[35] Zhu C (2021). Incidence and predictors of surgical site infection after distal femur fractures treated by open reduction and internal fixation: a prospective single-center study. BMC Musculoskelet Disord.

[36] Liu D (2020). Multiple preoperative biomarkers are associated with incidence of surgical site infection following surgeries of ankle fractures. Int Wound J.

[37] Liu GB (2018). Detection of serum procalcitonin and hypersensitive C-reactive protein in patients with pneumonia and sepsis. J Biol Regul Homeost Agents.

[38] Peng Q (2019). Expressions of plasma cystatin C, D-dimer and hypersensitive C-reactive protein in patients with intracranial progressive hemorrhagic injury after craniocerebral injury, and their clinical significance. Arq Neuropsiquiatr.

[39] Chen C (2021). Severe periodontitis is associated with the serum levels of hypersensitive C reactive protein and lipoprotein-associated phospholipase A2 in the patients of acute ischemic stroke. J Clin Neurosci.

[40] Yao A (2016). Clinical practice of procalcitonin and hypersensitive c-reactive protein test in neonatal infection. Pak J Pharm Sci.

